# Influence of Ethanol on Emulsions Stabilized by Low Molecular Weight Surfactants

**DOI:** 10.1111/1750-3841.14947

**Published:** 2019-12-16

**Authors:** Ana C. Ferreira, Antonio Sullo, Scott Winston, Ian T. Norton, Abigail B. Norton‐Welch

**Affiliations:** ^1^ School of Chemical Engineering Univ. of Birmingham Edgbaston B14 2TT UK; ^2^ Diageo Unit D Woodside Bishops Stortford CM23 5RG UK

**Keywords:** ethanol, droplet size, interfacial tension, oil‐in‐water emulsion, low molecular weight surfactant

## Abstract

**Abstract:**

The effect of ethanol on oil‐in‐water emulsions stabilized with low molecular weight surfactants was investigated. Oil‐in‐water emulsions were prepared containing varying percentages of ethanol and sunflower oil, and stabilized with different emulsifiers (Tween 20, Tween 80, and Lecithin). Droplet size, viscosity, density, and interfacial tension measurements were carried out. The droplet size of emulsions stabilized by each of the surfactants studied decreased with the addition of ethanol to the aqueous phase showing a minimum at a concentration of ethanol around 40%. The trend in droplet size is accompanied by a decrease in the interfacial tension between water and oil as the ethanol concentration increases. Viscosity measurements show that the change in viscosity of the final emulsion is the result of the change in viscosity of the continuous phase, as well as the change in solubility of the surfactants due to the addition of ethanol. The density of the continuous phase decreases with the addition of ethanol and it is possible to match the densities of the two phases in order to reduce the effect of creaming/sedimentation and improve stability. This study provides scientific evidence for the formulation of stable emulsions containing a range of ethanol form 0 to 40%.

**Practical Application:**

Formation and stability of food‐grade emulsions in the presence of ethanol.

## Introduction

Oil‐in‐water emulsions are ubiquitous dispersed systems present in a wide range of food products. Controlling the droplet size within an acceptable range is a key for the production of emulsion‐based products with desired properties such as texture, appearance, and stability.

In the case of alcoholic products, this requires an understanding of the effect of compositional parameters, such as surfactant type and concentration, as well as, the effect that ethanol has on the solvent properties of the continuous phase (Burgaud & Dickinson, 1990). Dickinson and Stainsby have described the binary system ethanol + water as a nonideal mixture from a thermodynamic point of view, due to the nonlinear changes of the physical properties of the mixture as more ethanol is present (Dickinson & Stainsby, 1987; Khattab, Bandarkar, Fakhree, & Jouyban, 2012). Properties, such as viscosity, surface tension, and density, for example, vary considerably with the addition of ethanol and, as a result, they have a significant impact on the preparation and stability of emulsions (McClements, 2015; Medina‐Torres, Calderas, Gallegos‐Infante, González‐Laredo, & Rocha‐Guzmán, 2009), so it is important to determine how these properties are affected by the presence of ethanol. Another important consideration is the phenomenon of Ostwald Ripening. This is a well‐known process that involves the growth of larger droplets at the expense of smaller droplets due to the mass transport of soluble disperse phase through the continuous phase. In systems containing high concentrations of ethanol, where the solubility of the oil in the aqueous phase increases, an increase in the ripening rate has been consistently reported (Dickinson & Golding, 1998; Dickinson, Ritzoulis, Yamamoto, & Logan, 1999; Espinosa & Scanlon, 2013; Radford, Dickinson, & Golding, 2004; Zeeb, Gibis, Fischer, & Weiss, 2012). It is not just the continuous phase per se that influences the stability of an emulsion, but its effect on the solubility of the emulsifier also needs to be considered (McClements, 2015). Various studies have been published on the interaction between ethanol and proteins (Agboola & Dalgleish, 1996; Dickinson, 1987; Dickinson & Golding, 1998; Dickinson & Woskett, 1988; Donnelly, 1987; Espinosa & Scanlon, 2013; Medina‐Torres et al., 2009), it is, therefore, well known that a change in the solvent quality can have enormous repercussions in protein‐stabilized emulsions (Agboola & Dalgleish, 1996; Dickinson & Woskett, 1988). As ethanol is a poor solvent for proteins (Dalgleish, 1997), above a certain concentration (usually 30 to 40% EtOH), the proteins will start to aggregate leading to precipitation, which in turn causes instability (Dickinson & Golding, 1998). However, not all reactions are adverse, in fact, in concentrations below that causing protein precipitation, ethanol can actually enhance emulsion stability (Dickinson & Woskett, 1988). It has been showed that the presence of ethanol reduces the interfacial tension between the continuous phase and the oil phase (Dickinson & Woskett, 1988) resulting in a smaller droplet size (Medina‐Torres et al., 2009), which according to Stokes’ equation improves the emulsion stability by retarding creaming/sedimentation rates (Espinosa & Scanlon, 2013). Other studies conducted using different types or different combinations of emulsifiers have shown that each emulsifier reacts differently to the presence of ethanol (Burgaud & Dickinson, 1990; Coupland, Brathwaite, Fairley, & McClements, 1997; Dickinson, Narhan, & Stainsby, 1989; Dickinson, Ritzoulis, & Povey, 1999; Xu, Nakajima, Nabetani, Iwamoto, & Liu, 2001). For instance, when both proteins and low molecular weight (LMW) surfactants are present in an emulsion it can lead to loss of stability (Dickinson et al., 1999). This is due to the displacement of the proteins by the surfactants, a process known as competitive destabilization (Wilde, Mackie, Husband, Gunning, & Morris, 2004). LMW surfactants are typically more surface active than proteins (Wilde et al., 2004), and at higher concentrations produce a lower interfacial tension (Dickinson et al., 1999), so they will compete with the proteins for interfacial area (Wilde et al., 2004). However, Dickinson et al. (1989) showed that, in the presence of ethanol, the combination of proteins with a “modest” amount of LMW surfactants can actually improve the stability of emulsions. Previously it has been suggested that in these conditions the ethanol and the LMW surfactant molecules formed the primary interfacial layer, and the proteins formed an adjacent secondary layer (Dickinson et al., 1989). More recently though, a different study has indicated that small molecule alcohols, such as ethanol, are only associated with the aqueous phase and do not accumulate at the interface (Coupland et al., 1997). However, to the authors’ best knowledge, no studies have been yet published on the effect of ethanol on simple emulsions stabilized solely with LMW surfactants. In this article, the effect of a range of ethanol concentrations (0 to 80%) on emulsion stabilized by food‐grade surfactant is investigated. To this end, two LMW surfactants—Tween 20 and Tween 80—were chosen for their close chemical structure. These two surfactants differ only in their hydrocarbon chain length, where Tween 80 has a longer chain than Tween 20. In order to have a more comprehensive view of the ethanol effect, a third surfactant, structurally different and also used in the food industry, was selected—Lecithin.

## Materials and Methods

### Materials

Ethanol (Absolute, ≥99.8%, analytical reagent grade) was purchased from Fisher Scientific (UK), Tween 20 and 80 were purchased from Sigma‐Aldrich Ltd (Gillingham, UK), and Lecithin (Refined) was purchased from Alfa Aesar (Lancashire, UK). Sunflower oil (SO) was purchased from the local supermarket. All materials were used with no further purification or modification. The water used in the preparation of all emulsions was passed through a reverse osmosis unit and then a milli‐Q water system.

### Emulsion preparation

Oil‐in‐water (O/W) emulsions were prepared in by combining a premixed continuous phase with sunflower oil (SO) on a Silverson L5M for 10 min at 4000 rpm (room temperature), with a fine emulsor screen. The continuous phase was prepared by mixing water, ethanol, and emulsifier, in the required percentages. All concentrations were calculated as a weight percentage (% W/W), and unless stated otherwise percentages always refer to the overall emulsion.

### Droplet size measurements

The droplet size was measured by a Malvern Mastersizer MS 2000 (Malvern Panalytical, UK) with a Hydro SM manual small volume sample dispersion unit attached. A refractive index of 1.33 for water and 1.467 for oil was used for the calculation of the droplet size distribution. For measurement, the sample was dispersed in distilled water at approximately 1300 rpm until an obscuration rate of 3 to 5% was achieved. The volume‐weighted mean diameter (D[4,3]) was obtained, and unless stated otherwise, droplet size always refers to this parameter. Samples were prepared in at least triplicates and are reported as the average of three measurements. Particle size measurements were taken immediately after preparation and after 10 months.

### Viscosity measurements

Viscosity measurements were performed using a Kinexus Pro Rheometer (Malvern Panalytical, UK), with double gap geometry using a shear rate profile from 0.1 to 100 s^−1^, at 25 °C. All measurements were performed in triplicates and an average value, as well as a standard deviation, was calculated.

### Density

Density was measured using a density set, consisting of a solid measuring probe (DE0601 – *ρ* = 2.330 g/cm^3^), on a K100 Kruss Tensiometer (Kruss GmbH, Germany). All measurements were performed in triplicates and the average value, as well as the standard deviation, was calculated.

### Interfacial measurements

Interfacial tension was measured on a K100 Tensiometer (Kruss GmbH, Germany), with the Wilhelmy plate method. The plate was immersed in the higher density phase to a depth of 3 mm. Once the surface has been detected, an interface between the two phases was created by carefully pipetting the lower density phase over the higher density phase. The test was conducted over 3600 s at room temperature. All measurements were performed in triplicates and the average value, as well as the standard deviation, was calculated.

## Results and Discussion

### Droplet size

**Figure 1:**
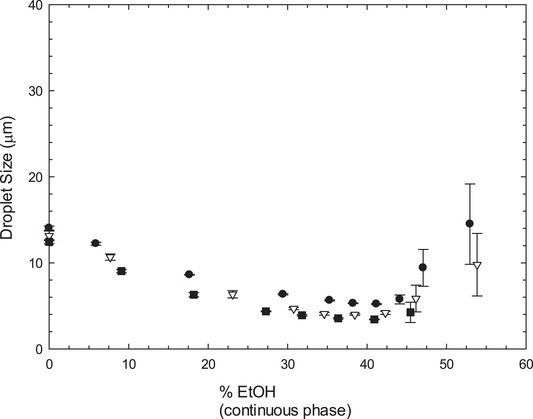
Comparison of the droplet size of emulsions prepared with •15, ∇35, and ■45% sunflower oil and 1% Tween 20, in the presence of varying percentages of ethanol (0‐55%). Emulsions were prepared on a high shear mixer and droplet size was measured by laser diffraction. Error bars represent one standard deviation; where not visible error bars are smaller than the symbols.

To understand the effect of ethanol (EtOH) on oil‐in‐water (O/W) emulsions stabilized by LMW surfactants, it is important to determine how it affects the droplet size, therefore, droplet size measurements were performed on samples containing varying percentages of ethanol with three different SO percentages—15, 35, and 45%. To avoid the complication of phase inversion, from oil‐in‐water (O/W) to water‐in‐oil (W/O) emulsion, samples with concentrations of oil higher than 50% were not considered in this study. The emulsions were stabilized with 1% Tween 20. Figure 1 shows that the droplet size decreases with ethanol concentration to then reach a minimum value around 40% ethanol. At concentrations of ethanol higher than 40%, each of the emulsions showed an increase in droplet size and eventually became unstable, which is the reason behind the high standard deviation reported for those samples. It was not possible to obtain results above 45% ethanol in the continuous phase for the formulations containing 45% SO, and due to the instability and consequent irreproducibility showed by the other formulations, results obtained above 45% ethanol in the continuous phase will not be used by the authors to draw any conclusions. For this reason, this discussion will focus on values below 45% ethanol. In Figure 1, it is possible to observe a significant decrease in the droplet size until the lowest values were obtained around 40% EtOH in the continuous phase, with values of 5.21 µm for 15% SO, 3.99 µm for 35% SO, and 3.43 µm for 45% SO (in systems without ethanol the values were 14.04, 13.14, and 12.36 µm, respectively). No significant changes can be observed between the different percentages of SO as the plots overlap, which leads the authors to believe that the decrease in droplet size and later instability is independent of SO content, but due to the presence of ethanol in the continuous phase.

**Figure 2:**
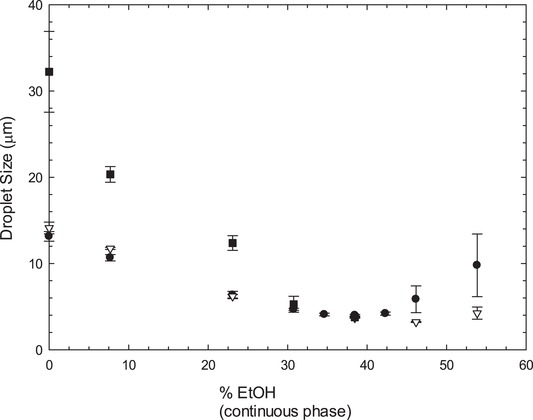
Comparison of the effect of three different LMW surfactants (1% Tween 20 •, Tween 80 ∇, and Lecithin ■) on the droplet size of emulsions prepared with varying percentages of ethanol (0‐55%) and 35% sunflower oil. Note that it was not possible to obtain results beyond 40% EtOH for Lecithin. Emulsions were prepared on a high shear mixer and droplet size was measured by laser diffraction. Error bars represent one standard deviation; where not visible error bars are smaller than the symbols.

In order to assert the effect of ethanol on the stability of emulsions stabilized with LMW surfactants, it was important to compare the results obtained with other food‐grade surfactants commercially available – Tween 80 and Lecithin. To this end, the formulations for 35% SO were repeated, but with 1% Tween 80 and 1% Lecithin as substitute surfactants, and the results compared with those of Tween 20 (see Figure 2). It was possible to observe that compared with the previously tested Tween 20, Tween 80 displays a similar behavior, with only a small shift in the trend, meaning that the lower droplet size (3.21 µm) was reached with a higher percentage of ethanol in the formulation, around 46%. As previously seen, after the smallest droplet size is achieved, the emulsions become increasingly unstable, as it is observed by the rapid increase in droplet size and the related large error bars. The emulsions stabilized with Lecithin showed a larger droplet size at lower percentages of ethanol when compared to Tween 20 and Tween 80, but reach a similar small size (3.76 µm) at around 40% ethanol in the continuous phase, like the other surfactants. At concentration of ethanol higher than 40%, emulsions become highly unstable and at 45% ethanol the droplet size obtained was around 70 µm, for this reason, Figure 2 does not include values for Lecithin above 40% ethanol. The effect of ethanol on the droplet size is partially explained by the change in the solvent properties of the aqueous phase as more water is replaced by ethanol. This alters the solubility of the surfactants monomers in aqueous solutions by making the aqueous phase less thermodynamically unfavorable for non‐polar groups, which impacts the partition of the surfactant at the interphase.

As the concentration of ethanol increases in the system, the difference among samples with different surfactants is less apparent and, finally, for concentrations of ethanol around 40%, the effect of ethanol appears to be dominant as all the samples exhibit the same droplet size. This suggests that the ethanol, at concentrations close to 40%, is able to penetrate into the surfactant monolayer causing a dramatic change in its optimum curvature and interfacial tension, both having an effect on reducing droplet size (Aramaki, Olsson, Yamaguchi, & Kunieda, 1999; Cavalli, Marengo, Caputo, Ugazio, & Gasco, 1996; Gradzielski, 1998; Yaghmur, Aserin, & Garti, 2002). Concentrations of ethanol higher than 40% cause Lecithin to precipitate, which explains why Lecithin seems to lose its functionality as an emulsifier for higher percentages of ethanol. Similarly, emulsions stabilized by Tween 20 and Tween 80, shown in Figure 2, become unstable at concentrations of ethanol higher than 40% because of the change in solubility. Both molecules become more soluble in the continuous phase and are, therefore, displaced from the interface.

### Viscosity

**Figure 3:**
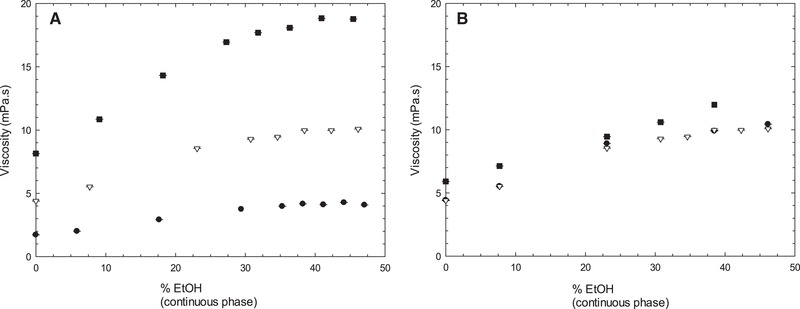
Comparison of viscosity of emulsion prepared with different percentages of ethanol: A with three different percentages of sunflower oil (•15, ∇35, and ■45%) and B prepared with 35% sunflower oil and stabilized with three different surfactants (1% Tween 20 ∇, Tween 80 •, and Lecithin ■). Emulsions were prepared on a high shear mixer and viscosity was measured on a rotational rheometer. Error bars represent one standard deviation; where not visible error bars are smaller than the symbols.

Viscosities were obtained for all the formulations prepared and, similarly to the individual components, all the emulsions prepared displayed a Newtonian behavior. Therefore, for comparison purposes and to better illustrate the effect of ethanol, a single shear rate was selected to plot against the ethanol percentage (Figure 3). The samples prepared with Tween 20 and different percentages of SO (Figure 3A) showed that the viscosity increases with the percentage of SO present. However, it was also possible to observe that within the same SO percentage, the viscosity increases with the percentage of ethanol present until it reaches around 45% EtOH in the continuous phase. At 0% ethanol, the viscosity values for different percentages of SO are closer together (≈2 to 8 mPa.s), whereas at 45% ethanol there was a greater variation in the values (≈4 to 19 mPa.s). A previous article published on the viscosity of the ethanol + water binary mixture (Khattab et al., 2012) shows that, at 25 °C, water has a viscosity of 0.89 mPa.s, the viscosity then increases with the addition of ethanol reaching values as high as ≈2.4 mPa.s at around 0.25 mole fraction of ethanol, which corresponds to 45% ethanol, before it decreases again until it reaches the viscosity for ethanol of 1.1 mPa.s. The change in viscosity of the aqueous phase plays a role in the overall viscosity, especially at low dispersed phase volume. In the case of the samples containing 45% of SO, the effect ethanol has on the solubility of the surfactant and, therefore, the properties of the interfacial layer can change the droplets‐solvent or droplets‐droplets interaction, which results in higher viscosity (Figure 3A). Figure 3B shows a comparison between emulsions prepared with the same percentage of SO, but different emulsifiers. Tween 80 shows no difference from Tween 20, whereas Lecithin still follows the same trend but has a slightly higher viscosity. The formulations compared are the same, differing only in the surfactant used, and since Tween 20 and Tween 80 have very similar molecules, it was expected that the viscosities would be similar. Lecithin, however, shows a slightly higher viscosity especially at concentration of ethanol approaching 40%. At high concentrations of ethanol, Lecithin become less soluble and it starts to aggregate promoting flocculation of droplets, thus, resulting in an increase in viscosity. This is also confirmed by the observation of a Lecithin sediment when the concentration of ethanol reached 50%.

### Density

**Figure 4:**
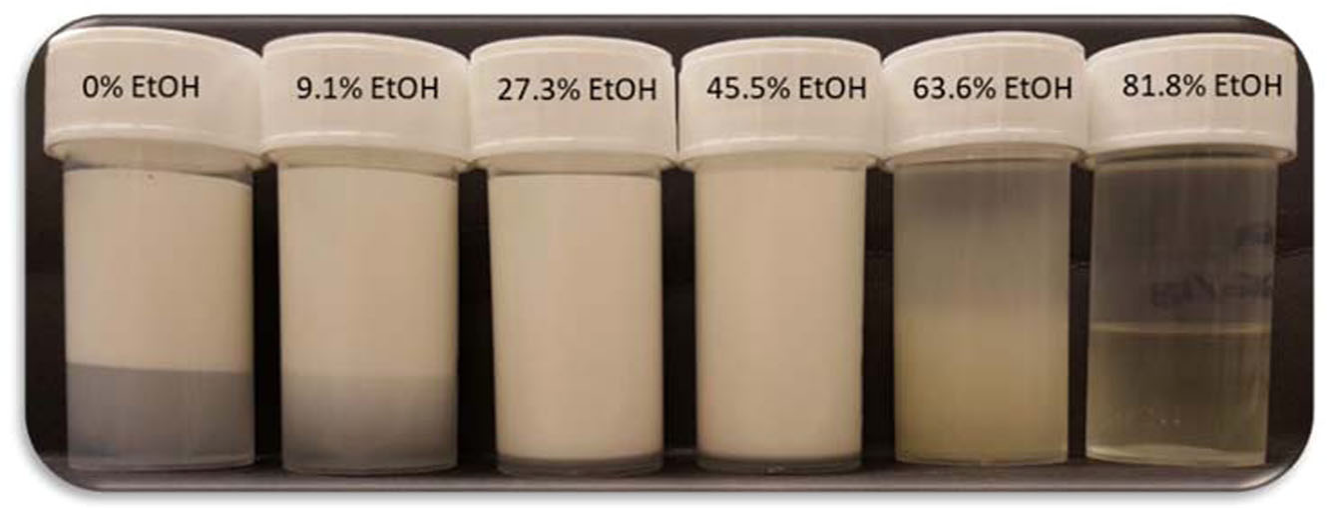
Emulsions containing varying percentages of ethanol, 3 days after preparation, showing different creaming and sedimentation rates.

**Figure 5:**
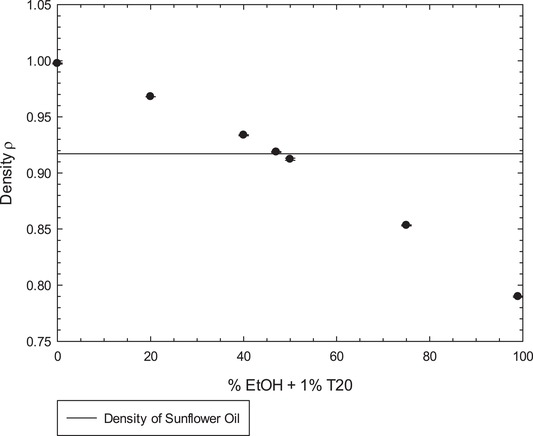
Graph of densities of different percentages of ethanol + water with 1% Tween 20, and comparison with density of sunflower oil. Error bars represent a single standard deviation; where not visible, error bars are smaller than the symbols.

Following the preparation of the emulsions, they were checked for instability. After a few hours or days, depending on the formulation, it was possible to observe that the formulations with lower percentages of ethanol showed creaming as expected, but the formulations with higher percentages of ethanol showed some sedimentation. It was also observed that some formulations, around 40% ethanol in the continuous phase showed much slower creaming/sedimentation (Figure 4). This appears to correlate with a change in the density of the continuous phase due to the presence of ethanol. As it has been demonstrated in a previous article (Khattab et al., 2012), the density of a water + ethanol mixture changes depending on the percentage of ethanol present in the mixture. At a certain percentage of ethanol, where the density of the continuous phase matches that of the oil droplets, it would seem plausible that the buoyancy would be eliminated and the droplets would remain suspended in the continuous phase. This reduction in creaming/sedimentation would, in turn, lead to less coalescence and more stable emulsions (McClements, 2015). To support this claim, density measurements of different percentages of ethanol and water, with 1% Tween 20, were obtained and compared with the density of SO. As shown in Figure 5, the density of the continuous phase decreases with the addition of ethanol, reaching a value close to the density of the SO at approximately 47% EtOH. The value measured at this point was 0.919, very close to the value of SO measured, which was 0.917.

### Interfacial tension

**Figure 6:**
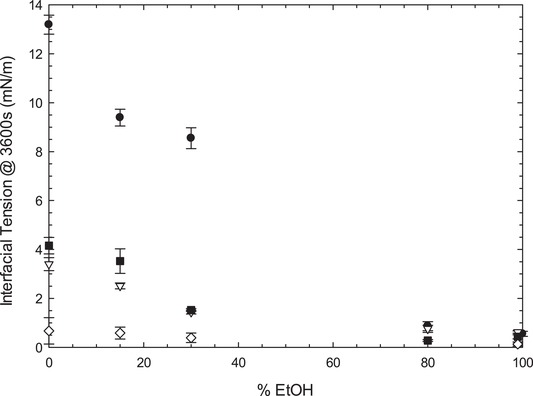
Comparison of interfacial tension between water and oil, in the presence of 1% Tween 20 ∇, 1% Tween 80 ■, 1% Lecithin, ◊ and in the absence of surfactant •. Due to density similarities, it was not possible to obtain the interfacial values between 30 and 80% EtOH. Error bars represent one standard deviation; where not visible error bars are smaller than the symbols.

It was suggested that the decrease in droplet size for ethanol percentages up to 40 to 45% was due to a decrease in the interfacial tension caused by the presence of ethanol in the continuous phase. In order to confirm this, a study of the interfacial tension of the systems was carried out (Figure 6). However, the techniques to measure interfacial tension currently available and used in this work, rely on differences of density to create the interface (Drelich, Fang, & White, 2015). As shown in the previous section of this article, the density of certain percentages of ethanol + water can be very similar to the density of SO. So due to the close values of density between both phases, it was not possible to obtain the values of interfacial tension between 30 and 80% ethanol. Literature values for the interfacial tension between SO and water vary between 21.2 to 27 mN/m (De Feijter, Benjamins, & Tamboer, 1987; Dragosavac, Sovilj, Kosvintsev, Holdich, & Vladisavljević, 2008; Fisher, Mitchell, & Parker, 1985; Mousavichoubeh, Shariaty‐Niassar, & Ghadiri, 2011; Santana, Perrechil, & Cunha, 2013; Xu, Nakajima, Nabetani, Ichikawa, & Liu, 2001), decreasing to approximately 5 mN/m in the presence of small nonionic surfactants like Tween 20 and Tween 80 (Dragosavac et al., 2008; Gomes, Costa, & Cunha, 2018; Kothekar, Ware, Waghmare, & Momin, 2007; Lloyd, Norton, & Spyropoulos, 2014; Mousavichoubeh et al., 2011; Santana et al., 2013). The literature value of interfacial tension of water/oil (LCT) with Lecithin is even lower at 1.2 mN/m (Ushikubo & Cunha, 2014). The values found in the literature for interfacial tension between ethanol and SO report an interfacial tension of approximately 2.33 mN/m (Duangsuwan, Tuzun, & Sermon, 2011; Duangsuwan, Tüzün, & Sermon, 2009; Xu et al., 2001) in the absence of surfactant. In the presence of small nonionic surfactant, the value of interfacial tension between ethanol and SO found in literature was 1.76 mN/m (Xu et al., 2001). It was not possible to find a value for interfacial tension between ethanol and SO in the presence of Lecithin in the current literature available. Nevertheless, commercially available unrefined oils often show a lower interfacial tension due to impurities and small amounts of naturally occurring surfactants present in the oils (Kralova & Sjöblom, 2009). For this reason, the interfacial tension measured by the authors were consistently lower than the values mentioned above, but it was still possible to identify a noticeable decrease in interfacial tension as the percentage of ethanol increases (Figure 6), which correlates with the decrease in droplet size shown in Figure 1. As shown in Figure 6, in the absence of ethanol, emulsions show different interfacial tension based on the type of surfactant used. As the concentration of ethanol increases, the interfacial tension for sample containing Tween decreases, which explains the decrease in droplet size observed. The effect of ethanol on the interfacial tension of samples containing Lecithin is much less apparent, which indicates that other effects such as the rate of adsorption of the surfactant at the interface need to be considered. At high percentages of ethanol, the surfactant does not seem to be having an effect. In fact, above 80%, the interfacial tension values with surfactant are very similar to the values obtained without surfactant. This leads the authors to conclude that above a certain value of ethanol, the decrease in interfacial tension is dominated by the presence of ethanol at the interphase.

### Long‐term stability—Droplet size after 10 months

Following their preparation, samples of the emulsions prepared with Tween 80 and Lecithin were kept at room temperature for 10 months.

**Figure 7:**
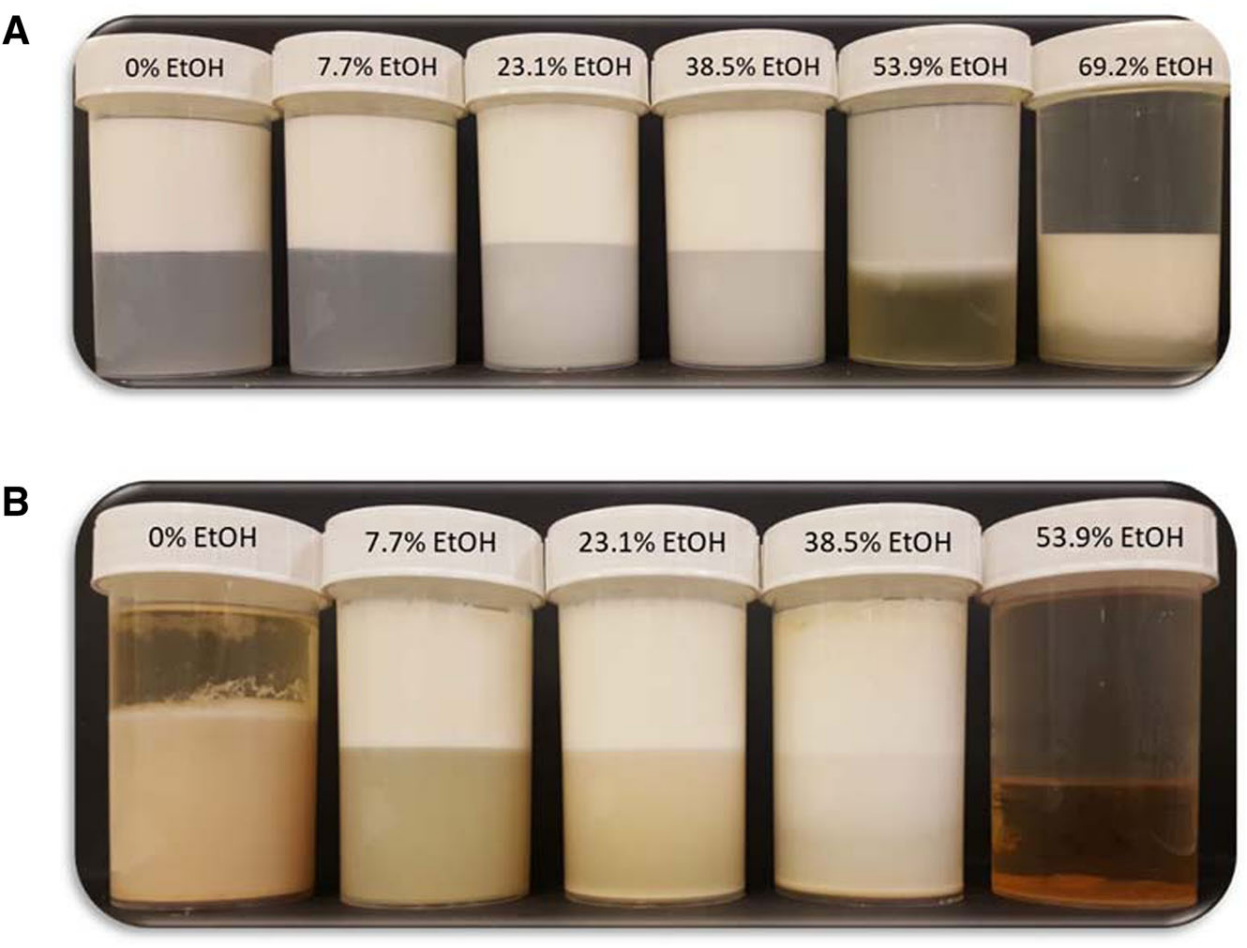
Emulsions containing different percentages of ethanol and stabilized with (A) Tween 80 and (B) Lecithin, 10 months after preparation.

**Figure 8:**
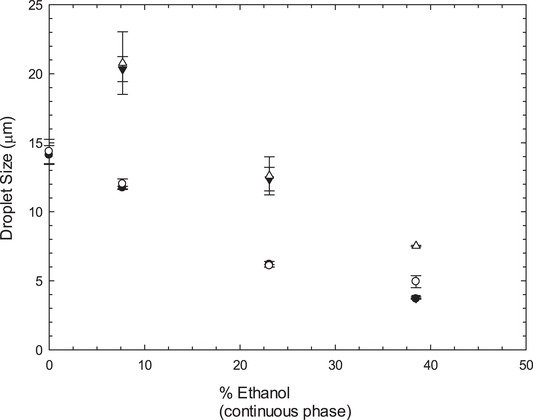
Comparison of droplet size of just prepared (closed symbols) and 10 months old (open symbols) emulsions, circles represent 1% Tween 80 and triangles represent 1% Lecithin. Error bars represent one standard deviation; where not visible error bars are smaller than the symbols.

As it is possible to observe from Figure 7A, emulsions stabilized with Tween 80 showed different levels of creaming up to about 40% ethanol in the continuous phase. This was expected considering the density differences between the dispersed and the continuous phase. Above 40% ethanol, however, it was possible to observe extensive destabilization, in the form of phase separation. Droplet size measurements of the not phase separated emulsions revealed that, although the emulsions creamed they were otherwise stable (Figure 8). Up to approximately 25%, there was no sign of coalescence or other forms of destabilization apart from creaming, as the droplet size was the same for the fresh and 10‐month old emulsions. At 38.5% ethanol, there was a small increase in droplet size that could be associated with Ostwald ripening due to the high ethanol content. Above 40% ethanol, it was not possible to measure droplet size as the samples had started to phase separate. It is thought that the increased solubility of Tween, due to the change in of polarity of the aqueous phase with the addition of ethanol, as well as, the presence of ethanol molecules at the interphase acting as co‐surfactants results in the displacement of the Tween form the interface.

In the case of Lecithin, in the absence of ethanol, it was not possible to form a stable emulsion (Figure 7B). Emulsions containing up to 40% ethanol, showed some creaming but appeared otherwise stable, however, above 40% the samples were completely phase separated. Droplet size measurements of the not phase separated emulsions revealed that for lower percentages of ethanol (7.7% and 23.1%), the emulsions were stable and showed no signs of destabilization apart from creaming, as the droplet size remained the same for fresh and for 10 months old emulsions (Figure 8). At 38.5% ethanol, however, there was an increase in droplet size that is likely to be caused by Ostwald ripening due to the high contents of ethanol. At concentrations of ethanol higher than 40%, samples were completely phase separated. At such concentrations, Lecithin becomes insoluble and eventually precipitates as observed in samples containing 53.9% of ethanol (Figure 7B).

## Conclusions

This study has provided additional evidence on the effect of ethanol on the formation and stability of oil‐in‐water emulsions stabilized by LMW surfactant. The presence of ethanol in the aqueous phase significantly decreases the droplet size of the emulsion, which reaches a minimum at a concentration of ethanol around 40%. The change in droplet size is mirrored by the change in interfacial tension. The effect to ethanol is rationalized in terms of changes in the solvent properties of the aqueous phase and its impact on the solubility of surfactant monomers in aqueous solutions and, consequently, its partition at the interphase.

Despite the different types of surfactant used, emulsions showed a similar droplet size at 40% ethanol suggesting that the ethanol at concentrations close to 40% may be present in the surfactant monolayer causing a dramatic change in its optimum curvature, and interfacial tension. This is also supported by the fact that ethanol on its own is capable of reducing interfacial tension. At concentrations above 40% ethanol, all emulsions become unstable and the interfacial tension is the same as samples containing only ethanol, indicating that the surfactants were essentially displaced of the interface. Nonetheless, under 40% ethanol the emulsions proved to be stable. In fact, long‐term stability tests have shown that, except for some creaming, the emulsions remained stable after 10 months, with no change in droplet size.

## Author Contributions

Ferreira collected, analyzed, and interpreted the data and drafted the manuscript. A. Sullo and S. Winston contributed to the conception and design of the work, revised the manuscript, and approved the final version to be published. I. Norton and A. Norton‐Welch contributed to the conception and design of the work, assisted in the interpretation of the data and revised the manuscript and approved the final version to be published. All authors agree to be accountable for all aspects of the work.

## Abbreviations

O/W: oil‐in‐water
W/O: water‐in‐oil
LMW: low molecular weight surfactant
SO: sunflower oil
EtOH: ethanol

## Conflict of Interest

The authors have no conflict of interest to declare.
